# Long-term survival and costs following extracorporeal membrane oxygenation in critically ill children—a population-based cohort study

**DOI:** 10.1186/s13054-020-02844-3

**Published:** 2020-04-06

**Authors:** Shannon M. Fernando, Danial Qureshi, Peter Tanuseputro, Sonny Dhanani, Anne-Marie Guerguerian, Sam D. Shemie, Robert Talarico, Eddy Fan, Laveena Munshi, Bram Rochwerg, Damon C. Scales, Daniel Brodie, Kednapa Thavorn, Kwadwo Kyeremanteng

**Affiliations:** 1 Division of Critical Care, Department of Medicine, University of Ottawa, Ottawa, ON Canada; 2 Department of Emergency Medicine, University of Ottawa, Ottawa, ON Canada; 3 ICES, Toronto, ON Canada; 4 Clinical Epidemiology Program, Ottawa Hospital Research Institute, Ottawa, ON Canada; 5 Bruyere Research Institute, Ottawa, ON Canada; 6 School of Epidemiology and Public Health, University of Ottawa, Ottawa, ON Canada; 7 Division of Palliative Care, Department of Medicine, University of Ottawa, Ottawa, ON Canada; 8 Department of Pediatrics, University of Ottawa, Ottawa, ON Canada; 9 Division of Critical Care, Children’s Hospital of Eastern Ontario, Ottawa, ON Canada; 10 Interdepartmental Division of Critical Care Medicine, University of Toronto, Toronto, ON Canada; 11 Department of Critical Care Medicine, The Hospital for Sick Children, Toronto, ON Canada; 12 Department of Pediatrics, McGill University, Montreal, QC Canada; 13 Division of Critical Care, Montreal Children’s Hospital, Montreal, QC Canada; 14 Toronto General Hospital Research Institute, University Health Network, Toronto, ON Canada; 15 Department of Medicine, Sinai Health System, Toronto, ON Canada; 16 Department of Medicine, Division of Critical Care, McMaster University, Hamilton, ON Canada; 17 Department of Health Research Methods, Evidence, and Impact, McMaster University, Hamilton, ON Canada; 18 Department of Critical Care Medicine, Sunnybrook Health Sciences Centre, Toronto, ON Canada; 19 Li Ka Shing Knowledge Institute, St. Michael’s Hospital, Toronto, ON Canada; 20 Division of Pulmonary, Allergy, and Critical Care Medicine, Department of Medicine, Columbia University College of Physicians and Surgeons and New York-Presbyterian Hospital, New York, NY USA; 21 Institut du Savoir Montfort, Ottawa, ON Canada

**Keywords:** Extracorporeal membrane oxygenation, ECMO, Pediatrics, Health services, Acute respiratory distress syndrome, Cardiogenic shock, Cardiac arrest

## Abstract

**Background:**

Extracorporeal membrane oxygenation (ECMO) is used to provide temporary cardiorespiratory support to critically ill children. While short-term outcomes and costs have been evaluated in this population, less is known regarding long-term survival and costs.

**Methods:**

Population-based cohort study from Ontario, Canada (October 1, 2009 to March 31, 2017), of pediatric patients (< 18 years of age) receiving ECMO, identified through the use of an ECMO procedural code. Outcomes were identified through linkage to provincial health databases. Primary outcome was survival, measured to hospital discharge, as well as at 1 year, 2 years, and 5 years following ECMO initiation. We evaluated total patient costs in the first year following ECMO.

**Results:**

We analyzed 342 pediatric patients. Mean age at ECMO initiation was 2.9 years (standard deviation [SD] = 5.0). Median time from hospital admission to ECMO initiation was 5 days (interquartile range [IQR] = 1–13 days). Overall survival to hospital discharge was 56.4%. Survival at 1 year, 2 years, and 5 years was 51.5%, 50.0%, and 42.1%, respectively. Among survivors, 99.5% were discharged home. Median total costs among all patients in the year following hospital admission were $147,957 (IQR $70,571–$300,295). Of these costs, the large proportion were attributable to the inpatient cost from the index admission (median $119,197, IQR $57,839–$250,675).

**Conclusions:**

Children requiring ECMO continue to have a significant in-hospital mortality, but reassuringly, there is little decrease in long-term survival at 1 year. Median costs among all patients were substantial, but largely reflect inpatient hospital costs, rather than post-discharge outpatient costs. This information provides value to providers and health systems, allowing for prognostication of short- and long-term outcomes, as well as long-term healthcare-related expenses for pediatric ECMO survivors.

## Introduction

Extracorporeal membrane oxygenation (ECMO) provides cardiorespiratory support to patients who have failed initial, conventional treatment [1, 2]. In pediatric populations, ECMO is used for organ support in cases of respiratory failure, cardiac failure, and as an adjunct to cardiopulmonary resuscitation (E-CPR) during cardiac arrest [3–6]. ECMO can also serve as a bridge to selected medical or surgical therapies, including ventricular assist device (VAD), and heart or lung transplant [7, 8].

With the use of ECMO evolving [9], there is a growing need to understand its impact on long-term patient outcomes and system cost. The majority of evidence surrounding the efficacy of ECMO in the pediatric population comes from institutional cohort studies, as very few randomized trials have been performed [2]. Across the world, ECMO utilization was historically more common among neonates than older children; however, this trend has changed over the last decade with an increase in pediatric cardiac and pediatric respiratory utilization [9]. For patients with respiratory failure, in-hospital mortality among those receiving ECMO (largely venovenous [VV-ECMO]) was approximately 25% and did not differ from propensity-matched controls [10]. However, in the pediatric population, venoarterial ECMO (VA-ECMO) is more common. Hospital mortality in this population has been reported between 45 and 55% [9].

ECMO has been used for critically ill children in Canada since 1989, but little has been published regarding longer-term outcomes or associated costs, and this has been identified as an important avenue for future research in extracorporeal life support [11, 12]. Most importantly, there are key gaps in knowledge related to costs incurred in the long-term. We conducted a population-based cohort analysis to evaluate the short- and long-term outcomes and costs of critically ill children receiving ECMO for cardiorespiratory support. We hypothesized that while many ECMO patients may die in hospital, the large majority who survived to discharge would still be alive at 5 years and that patient costs would largely reflect inpatient costs during the index hospitalization.

## Methods

### Data sources and setting

This was a population-based, retrospective cohort study, conducted using health administrative databases in Ontario, Canada. Ontario has a population of approximately 13 million inhabitants, and necessary medical services are publicly funded through a single-payer healthcare system. These databases record all medically necessary healthcare services, as well as physician, hospital, and demographic information for provincial residents. These data are held and linked at ICES, an independent, non-profit custodian of health data. ICES is funded by an annual grant from the Ontario Ministry of Health and Long-term Care. In Ontario, pediatric ECMO is provided at only two centers: The Hospital for Sick Children (Toronto, ON) and the Children’s Hospital of Eastern Ontario (Ottawa, ON). These institutions are regional, academic, university-affiliated children’s hospitals with cardiothoracic surgery programs. ECMO care in children is offered across Canada in similarly structured provincial pediatric ECMO centers. Cannulation procedures are conducted by surgeons (pediatric cardiothoracic or general surgeons), and medical/cardiac intensive care is centralized in “closed” units with pediatric intensive care physicians with training in ECMO. Bedside care involves a mix of speciality trained critical care nurses, respiratory therapists, ECMO specialists or perfusionists, and other allied health professionals.

We linked the following databases at the individual level using healthcare identification numbers: (1) The Canadian Institute for Health Information Discharge Abstract Database, (2) The Ontario Health Insurance Plan (OHIP) Claims Database, (3) The Registered Persons Database, (4) The Home Care Database, (5) The National Ambulatory Care Reporting System, (6) The National Rehabilitation Reporting System, (7) The Continuing Care Reporting System, and (8) Statistics Canada Census data.

This study was approved by The Ottawa Health Science Network Research Ethics Board.

### Patients

We included all pediatric patients (< 18 years of age) receiving ECMO in Ontario between October 1, 2009, and March 31, 2017. Patients were identified using the presence of an inpatient ECMO intervention code, obtained by searching through inpatient data captured by the Discharge Abstract Database (all codes displayed in Supplemental Table 1), as performed previously in adult patients [13].

We identified pediatric complex chronic conditions (CCC) from our administrative data using the methodology described by Feudtner et al. [14]. The CCC is preferentially used to identify comorbidities in children. We used the same codes as previous studies identifying CCC from the ICES administrative databases [15].

Outcomes following ECMO initiation were expected to be strongly associated with the indication (cardiac versus respiratory failure) [16], and therefore, we further analyzed patients on this basis. Since data related to ECMO mode (e.g., VV-ECMO vs. VA-ECMO) were unavailable, patients were categorized as either “Respiratory Failure” or “Cardiac Failure,” based upon the ICD-10 most responsible diagnosis (see Supplemental Table 1 for categorization). These categories were chosen in order to align with the Extracorporeal Life Support Organization (ELSO) database. Categorization was performed by two pediatric intensivists who also serve as medical directors for ECMO at the two pediatric centers in Ontario that provide this therapy (SD, AMG). Neonatal patients (0–30 days of age) were analyzed separately.

### Outcomes

The primary outcome was survival, which was measured to hospital discharge, as well as at 7 days, 30 days, 1 year, 2 years, and 5 years following initiation of ECMO. Time to ECMO initiation was estimated by calculating the difference between the hospital admission date and the date on which the ECMO intervention code was coded. Length of stay for the hospitalization was reported from the date of hospital admission to the date of discharge, or in-hospital death. Discharge disposition was determined using a hierarchy approach (Supplemental Table 3). Transplant and VAD implantation (short- or long-term) were determined using the relevant procedural codes in the Discharge Abstract Database.

Among this cohort, we examined the total and sector-specific direct healthcare costs accumulated in the year following the date of the index hospital admission (including the admission itself). We obtained all records of healthcare use paid for by the Ontario Ministry of Health and Long-term Care following admission. We estimated the costs associated with each record using previously described methods developed for health administrative data [17]. We used a top-down approach through case-mix methodology for sectors that use global budgets (e.g., hospital, complex continuing care, rehabilitation). Sectors for which each use has an associated fee payment (e.g., drug costs, physician remuneration) have costs estimated directly. All costs were expressed in 2018 Canadian dollars, and past costs were inflated using the healthcare-specific yearly Consumer Price Index reported by Statistics Canada.

Cell sizes with ≤ 5 patients are suppressed, as per ICES regulations, to protect patient confidentiality, though data were still analyzed.

### Statistical analyses

All statistical analyses were conducted using SAS Enterprise Guide 7.1 (SAS Institute Inc., Cary, NC). We present data as mean values, with standard deviation (SD), or medians, with interquartile range (IQR), where appropriate. Student’s *t* test (parametric values), Mann-Whitney test (non-parametric values), and *χ*^2^ (for categorical values) were performed to determine between-group differences. The Kaplan-Meier survival curves were used to measure trends of 1-year survival.

## Results

We included a total of 342 patients in the analysis. Of these, 103 (30.1%) were neonatal (0–30 days old), 169 (49.4%) were categorized as “Cardiac Failure,” and 70 (20.5%) were categorized as “Respiratory Failure.” Patient characteristics of the study cohort are shown in Table 1. Mean age was 2.9 years (SD 5.0 years), and 54.7% were male. Of the total patient cohort, 25.4% came from the lowest income quintile, compared with 14.3% from the highest income quintile. Median time to ECMO initiation from the date of hospital admission was 5 days (IQR 1–13 days). At least one CCC was present in 274 patients (79.9%), with the most common CCC being cardiovascular, which was present in 217 patients (63.3%).

**Table 1:** Characteristics of pediatric patients requiring extracorporeal membrane oxygenation in Ontario, Canada (2009–2016)

Variable	Overall (*n* = 342)	Neonatal (*n* = 103)	Cardiac failure (*n* = 169)	Respiratory failure (*n* = 70)
Sex, *n* (%)				
Male	187 (54.7)	66 (64.1)	79 (46.7)	42 (60.0)
Female	155 (45.3)	37 (35.9)	90 (53.3)	28 (40.0)
Age, years (non-neonatal) or days (neonatal), mean (SD)	2.9 (5.0)	9.6 (7.6)	3.0 (4.7)	7.0 (6.2)
Income, *n* (%)				
Lowest	87 (25.4)	33 (32.0)	37 (21.9)	17 (24.3)
Low	64 (18.7)	21 (20.4	36 (21.3)	7 (10.0)
Middle	72 (21.1)	22 (21.4)	35 (20.7)	15 (21.4)
High	67 (19.6)	15 (14.6)	33 (19.5)	19 (27.1)
Highest	49 (14.3)	12 (11.7)	25 (14.8)	12 (17.1)
Unknown	*	*	*	*
Rurality, *n* (%)				
Urban	305 (89.2)	93 (90.3)	148 (87.6)	64 (91.4)
Rural	35 (10.2)	10 (9.7)	19 (11.2)	6 (8.6)
Time to ECMO from admission, days, median (IQR)	5 (1–13)	5 (2–11)	5 (1–14)	6 (1–19)
Chronic complex conditions, *n* (%)^a^				
Any complex chronic condition	274 (79.9)	81 (78.6)	140 (82.8)	53 (74.6)
Prematurity	52 (15.2)	18 (17.5)	23 (13.6)	11 (15.5)
Cardiovascular	217 (63.3)	61 (59.2)	132 (78.1)	24 (33.8)
Metabolic	19 (5.5)	0 (0.0)	7 (4.1)	12 (16.9)
Other congenital or genetic abnormality	58 (16.9)	17 (16.5)	31 (18.3)	10 (14.1)

^a^Adapted from Feudtner et al. [14]. *Abbreviations*: *SD* standard deviation, *ECMO* extracorporeal membrane oxygenation, *IQR* interquartile range

Range provided due to small cell sizes

*Denotes ≤ 5 patients

**Fig. 1:**
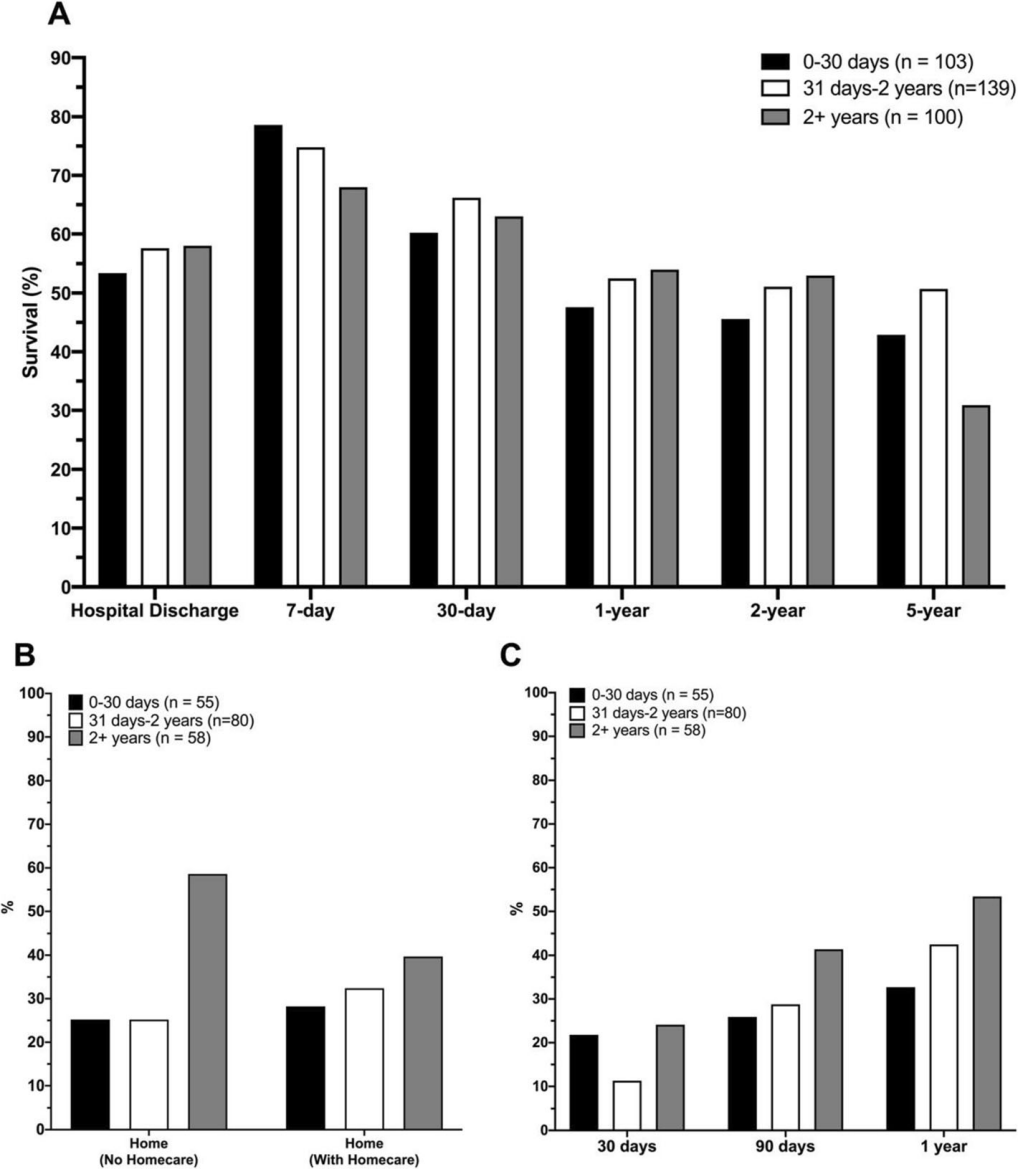
Outcomes of critically ill pediatric patients requiring extracorporeal membrane oxygenation in Ontario (*n* = 342), stratified by age bracket. **a** Mortality. **b** Disposition among patients surviving to hospital discharge. **c** Hospital readmission in the first year among patients surviving to hospital discharge

Table 2 shows patient outcomes for the entire cohort and by individual subgroups. A total of 193 patients (56.4%) survived to hospital discharge. The 1-year, 2-year, and 5-year survival for the cohort were 51.5%, 50.0%, and 42.1%, respectively. Survival to discharge was lower among respiratory failure patients (51.4%), as compared to cardiac failure patients (60.4%). Among neonatal patients, 53.4% survived to hospital discharge. Survival at 1 year, 2 years, and 5 years were 47.6%, 45.6%, and 42.9%, respectively. Median overall hospital length of stay was 26 days (IQR 11–61 days). Fifty-nine patients (17.3%) were transitioned from ECMO to VAD. ECMO was followed by heart transplant in 13 patients (3.8%) and lung transplant in 11 patients (3.2%). Among patients surviving to discharge, 192 (99.5%) were discharged home, though 97 of these patients (50.5%) required homecare. In the year following hospitalization, 57.5% of survivors had at least one Emergency Department visit, and 43.0% of survivors required hospital re-admission. Patient outcomes stratified by age are shown in Fig. 1.

**Table 2:** Short- and long-term outcomes of pediatric patients requiring extracorporeal membrane oxygenation in Ontario, Canada (2009–2017)

Variable	Overall (*n* = 342)	Neonatal (*n* = 103)	Cardiac failure (*n* = 169)	Respiratory failure (*n* = 70)	*P* value
Survival following ECMO initiation, *n* (%)					
To hospital discharge	193 (56.4)	55 (53.4)	102 (60.4)	36 (51.4)	0.34
7 days	253 (74.0)	81 (78.6)	127 (75.1)	45 (64.3)	0.10
30 days	217 (63.5)	62 (60.2)	115 (68.0)	40 (57.1)	0.20
1 years	176 (51.5)	49 (47.6)	93 (55.0)	34 (48.6)	0.42
2 years	171 (50.0)	47 (45.6)	91 (53.8)	33 (47.1)	0.37
5 years^a^	75 (42.1)	24 (42.9)	38 (45.2)	13 (34.2)	0.51
Hospital length of stay, days, median (IQR)	26 (11–61)	24 (14–53)	29 (11–72)	21 (8–55)	0.21
Ventricular assist device during hospitalization, *n* (%)	59 (17.3)	17 (16.5)	29 (17.2)	13 (18.6)	0.94
Transplant during hospitalization, *n* (%)					
Heart transplant	13 (3.8)	*	13 (7.7)	*	< 0.001
Lung transplant	11 (3.2)	*	*	10 (14.3)	< 0.001
Discharge disposition among survivors, *n* (%)^b^					0.78
Home (without homecare)	95 (49.2)	26 (47.2)	51 (50.0)	18 (50.0)	
Home (with homecare)	97 (50.3)	29 (52.7)	50 (49.0)	18 (50.0)	
Emergency department visit following discharge, *n* (%)^b^					
Within 30 days	48 (24.9)	14 (25.5)	23 (22.5)	11 (30.6)	0.90
Within 90 days	78 (40.4)	22 (40.0)	42 (41.1)	14 (38.9)	0.66
Within 1 year	111 (57.5)	27 (49.1)	58 (56.9)	26 (72.2)	0.25
Hospital readmissions following discharge, *n* (%)^b^					
Within 30 days	35 (18.1)	12 (21.8)	15 (14.7)	8 (22.2)	0.71
Within 90 days	62 (32.1)	15 (27.3)	33 (32.4)	14 (38.9)	0.53
Within 1 year	83 (43.0)	18 (32.7)	43 (42.2)	22 (61.1)	0.10

*Abbreviations*: *ECMO* extracorporeal membrane oxygenation, *IQR* interquartile range

^a^Five-year mortality values only include patients between 2009 and 2014 (*n* = 178 for “Overall,” *n* = 56 for “Neonatal,” *n* = 84 for “Cardiac failure,” *n* = 38 for “Respiratory failure”)

^b^Only includes patients who survived to hospital discharge (*n* = 193 for “Overall,” *n* = 55 for “Neonatal,” *n* = 102 for “Cardiac failure,” *n* = 36 for “Respiratory failure”)

*Denotes ≤ 5 patients

**Fig. 2:**
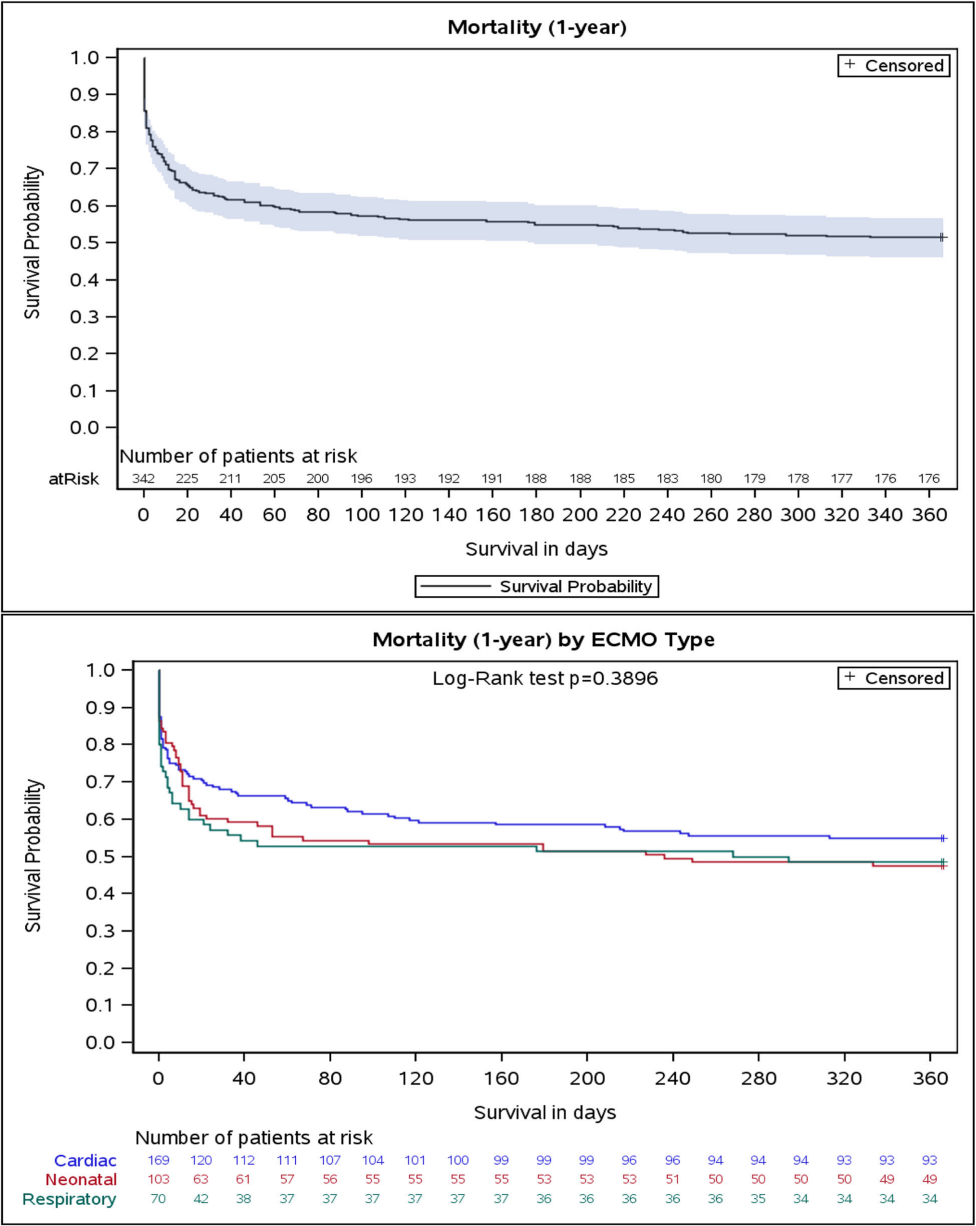
The Kaplan-Meier curves depicting survival in the first year following extracorporeal membrane oxygenation among critically ill pediatric patients in Ontario, Canada (2009–2017) (*n* = 342)

The Kaplan-Meier curves depicting survival in the first year following ECMO initiation for the entire cohort and by ECMO indication are shown in Fig. 2. The greatest proportion of deaths occurred in the first 30–40 days following ECMO initiation. We compared patients who died in-hospital with those that survived to discharge (Supplemental Tables 5, 6, 7). A shorter median time to ECMO initiation was associated with survival among the “Neonatal” and “Cardiac Failure” groups.

Importantly, we evaluated median health system costs among pediatric patients receiving ECMO in the 1-year following hospital admission (including the admission itself) (Table 3). Median total costs among all patients were $147,957 (IQR $70,571–$300,295). The predominant contributor to overall costs was inpatient costs of the index hospitalization (median $119,197, IQR $57,839–$250,675). Mean costs are shown in Supplemental Table 8.

**Table 3:** Median (interquartile range) 1-year costs following admission of pediatric patients requiring extracorporeal membrane oxygenation in Ontario, Canada (2009–2017)

Cost sector	Overall (*n* = 342)	Neonatal (*n* = 103)	Cardiac failure (*n* = 169)	Respiratory failure (*n* = 70)	*P* value
Acute care sectors					
Inpatient	119,197 (57,839–250,675)	117,885 (66,597–217,202)	135,879 (61,181–289,533)	96,762 (41,416–194,397)	0.15
Emergency department	143 (0–807)	0 (0–698)	111 (0–806)	475 (0–965)	0.04
Continuing care sectors					
Complex continuing care	0 (0–0)	0 (0–0)	0 (0–0)	0 (0–0)	0.60
Long-term care	0 (0–0)	0 (0–0)	0 (0–0)	0 (0–0)	1.00
Rehabilitation	0 (0–0)	0 (0–0)	0 (0–0)	0 (0–0)	1.00
Homecare	0 (0–1734)	0 (0–1628)	0 (0–1636)	0 (0–2633)	0.92
Outpatient care sectors					
Outpatient clinics	758 (319–2307)	722 (0–2287)	780 (332–1946)	737 (0–3510)	0.84
Laboratory (OHIP)	0 (0–0)	0 (0–0)	0 (0–0)	0 (0–0)	0.07
Drugs (Ontario Drug Benefit Program)	0 (0–548)	0 (0–224)	0 (0–804)	0 (0–1183)	0.66
Physician billings	18,970 (10,059–31,348)	17,649 (12,106–24,460)	21,529 (11,492–34,973)	17,902 (7209–28,076)	0.19
Total costs	147,957 (70,571–300,295)	141,423 (84,755–256,128)	173,303 (81,888–328,018)	147,957 (70,571–300,295)	0.29
Total cost/survivor to discharge	412,358	365,393	412,284	484,328	

All values in Canadian dollars. *Abbreviations*: *SD* standard deviation, *OHIP* Ontario Health Insurance Plan

## Discussion

We conducted a population-based cohort study among critically ill children receiving ECMO for cardiorespiratory support to identify long-term outcomes and health system costs in the first year. Survival to hospital discharge in this provincial-wide cohort was 56.4%. This did not decrease substantially after 1 year, but moderate decrease was seen at 2 years and 5 years following ECMO initiation. The number of patients in the cohort from the lowest income quintile was nearly double that of patients from the highest income quintile. Patients predominantly received ECMO for cardiac failure, and survival to hospital discharge was markedly higher in this population, as compared to those with respiratory failure. The large majority of survivors to discharge (99.6%) were ultimately able to be discharged home. Finally, pediatric patients accrued significant hospital-related costs in the year following ECMO initiation, but the majority of these costs were incurred during the inpatient hospitalization, with few costs incurred following discharge. This study provides important information regarding long-term survival and costs following the use of this high-intensity therapy in the care of critically ill pediatric patients.

This cohort represents a contemporary pediatric ECMO sample that spans over 7 years. In-hospital mortality in this overall cohort was similar to that reported in other studies [9]. Reassuringly, there was relatively little mortality following hospital discharge, and survival rates at 1 year were comparable to hospital discharge rates. Survival at 2 years and 5 years following initiation of ECMO showed a moderate decline. Similar 5-year trends have been previously demonstrated in a population-based pediatric cohort from the UK [18] and an adult cohort from Ontario [13]. Therefore, this study supports existing work showing that particularly in patients > 30 days of age, the long-term survival (up to 5 years) following ECMO is very good among those who survive to hospital discharge. This data has particular value in conversations with patients and caregivers, and can be used to convey prognostication and set expectations for survival in both the short- and long-term, given the existing data. Overall mortality rates were higher among patients with respiratory failure, which is contrary to what has been seen previously [8–10]. Although the reason for this remains unclear, it may reflect the selective use of ECMO at the included centers, as compared to other centers in the ELSO database. For this reason, caution must be exercised in extrapolating the findings from the subgroup analyses to other settings.

Few pediatric patients received heart or lung transplantation following ECMO initiation, which is in large contrast to what is seen in adult patients [13]. This is in keeping, however, with the practice in pediatrics that has shifted away from using ECMO as a bridge to transplantation, and replaced it by using VAD, given the better outcomes with VAD over ECMO [19, 20]. Data from the ELSO Registry shows that among patients for whom ECMO was planned as a bridge to heart transplant, only 45% ultimately survived to transplantation, and a third of transplanted patients died before hospital discharge [19].

Importantly, we investigated long-term costs following utilization of ECMO in critically ill children. While there is emerging literature on the cost-effectiveness of ECMO [21, 22], and hospital costs for pediatric patients receiving ECMO have been previously published [21, 23–25], our study uniquely describes long-term costs in the year following ECMO initiation. We found that patients supported with ECMO accrued significant costs in the year following their index hospitalization; however, the large majority of these costs reflect the ECMO hospitalization itself, and few costs were incurred following discharge. The median overall costs in our population are similar to what has been previously described among pediatric patients with cardiorespiratory failure [26]. These costs are also consistent with those seen in other high-resource critically ill populations, such as pediatric patients with traumatic brain injury [27], or adults with multi-organ failure [28]. A major focus of current critical care research is the understanding of resource utilization not only in the short-term, but also following discharge [29, 30]. ECMO particularly falls into this conversation given the significant amount of resources required for its use. Our study shows that while the inpatient costs are significant, the downstream costs are less so, and this has particular importance in healthcare settings where downstream costs are paid for by the patients themselves.

Finally, our work identifies important avenues for future research, especially related to patient outcomes, and selection for ECMO. We compared and contrasted factors associated with survival among children receiving ECMO. We also demonstrated that ECMO was associated with significant inpatient costs. Therefore, to appropriately prognosticate outcome, and optimize patient selection, derivation and validation of a clinical decision instrument is necessary. Ideally, this should be performed using a prospective cohort, and separate instruments for patients with respiratory and cardiac failure should be derived, as has been done in the adult population [31, 32]. Furthermore, beyond understanding survival, an effort must be made to understand long-term quality of life, as has been done in the adult population [33]. For patients and caregivers, an appropriate picture of survival following ECMO must also include long-term functional and cognitive outcomes. While this has been performed at the 6-month point following ECMO [34, 35], longer-term follow-up is required.

In this study, we utilized comprehensive population-level data to investigate the short- and long-term outcomes and costs among critically ill pediatric patients receiving ECMO in Ontario, Canada. This study provides some of the first data related both to long-term outcomes and costs following the critical illness hospitalization in a large representative sample of Canadian pediatric ECMO patients. However, there are limitations to this study. First, ECMO is known to be associated with important complications, including cerebral hemorrhage, stroke, renal failure, and infection [36, 37]. We did not have validated data related to these complications and therefore are unable to comment on incidence and impact in our cohort. Second, in Ontario, ECMO in pediatric patients is conducted at only two specialized, regional referral centers for cardiac surgery, solid organ transplantation, trauma, and high-risk neonatal care. Since outcomes from ECMO are associated with center-specific frequency of use and volume [38], these findings are only generalizable to similar organizations with mature ECMO programs in North and South America, Europe, East Asia, Australia, and New Zealand. Third, while we did provide data related to survival and costs incurred following discharge, we did not have data related to quality of life and family burden following discharge. Existing evidence highlights the significant long-term morbidity seen in similar populations [39]. Finally, while the databases held at ICES are robust and have been used extensively for large population-based studies [13, 40], there are inherent limitations to the available administrative data. We did not have complete data related to illness severity at the time of hospital admission or ECMO initiation, nor the duration of ECMO. We also did not have data on parameters prior to ECMO initiation, configuration utilized, nor the specific costs attributable to ECMO use from a patient’s total inpatient costs. Lastly, we did not have data on mode or cause leading to death during the hospitalization or after discharge, the latter particularly pertinent among patients who initially survived to hospital discharge.

## Conclusions

We found that 55.5% of critically ill pediatric patients requiring ECMO survive to hospital discharge, with relative stabilization of the survival curve at 1, 2, and 5 years following ECMO initiation. Survival was lower among neonates than older children, and among patients with respiratory failure, as compared to cardiac failure. Reassuringly, in this contemporary cohort of children, nearly all patients surviving to hospital discharge were able to be discharged home. While ECMO patients accrued significant inpatient costs, these costs were less than typical high-cost populations, and most importantly, survivors had low healthcare-associated costs in the year following discharge. This work provides novel data regarding the long-term outcomes and costs of critically ill children receiving ECMO for cardiorespiratory support.

## Supplementary information

**Additional file 1 : Supplemental Table 1.** Relevant databases and codes utilized for ECMO identification, and International Classification of Diseases, Version 10 (ICD-10) diagnostic codes for categorization.

**Additional file 2 : Supplemental Table 2**. Ontario Health Insurance Plan (OHIP), International Classification of Diseases, Version 9 (ICD-9), and Version 10 (ICD-10) diagnostic codes for categorization of comorbidities.

**Additional file 3 : Supplemental Table 3.** Hierarchy approach for discharge disposition of those who survived to discharge.

**Additional file 4 : Supplemental Table 4.** Most common “Most Responsible Diagnoses” among included patients.

**Additional file 5 : Supplemental Table 5.** Comparison of neonatal patients receiving Extra Corporeal Membrane Oxygenation who survive to hospital discharge against those who died in-hospital (*n =* 342). *≤ 5 patients. ^a^Range provided due to small cell sizes. Abbreviations: SD = standard deviation; ECMO = Extracorporeal Membrane Oxygenation; IQR = interquartile range.

**Additional file 6: Supplemental Table 6.** Comparison of patients with cardiac failure receiving Extra Corporeal Membrane Oxygenation who survive to hospital discharge against those who died in-hospital (*n =* 169). *≤ 5 patients. ^a^Range provided due to small cell sizes. Abbreviations: SD = standard deviation; ECMO = Extracorporeal Membrane Oxygenation; IQR = interquartile range.

**Additional file 7 : Supplemental Table 7.** Comparison of patients with respiratory failure receiving Extra Corporeal Membrane Oxygenation who survive to hospital discharge against those who died in-hospital (*n =* 70). *≤ 5 patients. ^a^Range provided due to small cell sizes. Abbreviations: SD = standard deviation; ECMO = Extracorporeal Membrane Oxygenation; IQR = interquartile range.

**Additional file 8 : Supplemental Table 8.** Mean (standard deviation) 1-year costs following admission of pediatric patients requiring Extra Corporeal Membrane Oxygenation in Ontario, Canada (2009-2016). All values in Canadian dollars. Abbreviations: SD = standard deviation; OHIP = Ontario Health Insurance Plan.

## Data Availability

The datasets generated and analyzed are not publicly available due to patient privacy considerations and are under the jurisdiction of ICES.

## Abbreviations

CCI: Charlson Comorbidity Index
ECMO: Extracorporeal membrane oxygenation
ELSO: Extracorporeal Life Support Organization
IQR: Interquartile range
OHIP: Ontario Health Insurance Plan
SD: Standard deviation
VAD: Ventricular assist device
VA-ECMO: Venoarterial extracorporeal membrane oxygenation
VV-ECMO: Venovenous extracorporeal membrane oxygenation

## References

[1] MacLaren G, Dodge-Khatami A, Dalton HJ, MacLaren G, Dodge-Khatami A, Dalton HJ, Adachi I, Almodovar M, Annich G, Bartlett R (2013). Joint statement on mechanical circulatory support in children: a consensus review from the Pediatric Cardiac Intensive Care Society and Extracorporeal Life Support Organization. Pediatr Crit Care Med.

[2] Bembea MM, Hoskote A, Guerguerian AM (2018). Pediatric ECMO research: the case for collaboration. Front Pediatr.

[3] Bartlett RH, Roloff DW, Cornell RG, Andrews AF, Dillon PW, Zwischenberger JB (1985). Extracorporeal circulation in neonatal respiratory failure: a prospective randomized study. Pediatrics.

[4] O’Rourke PP, Crone RK, Vacanti JP, Ware JH, Lillehei CW, Parad RB, Epstein MF (1989). Extracorporeal membrane oxygenation and conventional medical therapy in neonates with persistent pulmonary hypertension of the newborn: a prospective randomized study. Pediatrics.

[5] Lasa JJ, Rogers RS, Localio R, Shults J, Raymond T, Gaies M, Thiagarajan R, Laussen PC, Kilbaugh T, Berg RA (2016). Extracorporeal cardiopulmonary resuscitation (E-CPR) during pediatric in-hospital cardiopulmonary arrest is associated with improved survival to discharge: a report from the American Heart Association’s get with the guidelines-resuscitation (GWTG-R) registry. Circulation.

[6] Bembea MM, Ng DK, Rizkalla N, Rycus P, Lasa JJ, Dalton H, Topjian AA, Thiagarajan RR, Nadkarni VM, Hunt EA (2019). Outcomes after extracorporeal cardiopulmonary resuscitation of pediatric in-hospital cardiac arrest: a report from the get with the guidelines-resuscitation and the extracorporeal life support organization registries. Crit Care Med.

[7] Hetzer R, Javier M, Delmo Walter EM (2018). Role of paediatric assist device in bridge to transplant. Ann Cardiothorac Surg.

[8] Lin JC (2017). Extracorporeal membrane oxygenation for severe pediatric respiratory failure. Respir Care.

[9] Barbaro RP, Paden ML, Guner YS, Raman L, Ryerson LM, Alexander P, Nasr VG, Bembea MM, Rycus PT, Thiagarajan RR (2017). Pediatric extracorporeal life support organization registry international report 2016. ASAIO J.

[10] Barbaro RP, Xu Y, Borasino S, Truemper EJ, Watson RS, Thiagarajan RR, Wypij D, Curley MAQ (2018). Does extracorporeal membrane oxygenation improve survival in pediatric acute respiratory failure?. Am J Respir Crit Care Med.

[11] Combes A, Brodie D, Chen YS, Fan E, Henriques JPS, Hodgson C, Lepper PM, Leprince P, Maekawa K, Muller T (2017). The ICM research agenda on extracorporeal life support. Intensive Care Med.

[12] Brodie D, Vincent JL, Brochard LJ, Combes A, Ferguson ND, Hodgson CL, Laffey JG, Mercat A, Pesenti A, Quintel M (2018). Research in extracorporeal life support: a call to action. Chest.

[13] Fernando SM, Qureshi D, Tanuseputro P, Fan E, Munshi L, Rochwerg B, Talarico R, Scales DC, Brodie D, Dhanani S (2019). Mortality and costs following extracorporeal membrane oxygenation in critically ill adults: a population-based cohort study. Intensive Care Med.

[14] Feudtner C, Feinstein JA, Zhong W, Hall M, Dai D (2014). Pediatric complex chronic conditions classification system version 2: updated for ICD-10 and complex medical technology dependence and transplantation. BMC Pediatr.

[15] Buchan SA, Chung H, Campitelli MA, Crowcroft NS, Gubbay JB, Karnauchow T, Katz K, McGeer AJ, McNally JD, Richardson D (2017). Vaccine effectiveness against laboratory-confirmed influenza hospitalizations among young children during the 2010-11 to 2013-14 influenza seasons in Ontario, Canada. PLoS One.

[16] Schmidt M, Brechot N, Combes A (2016). Ten situations in which ECMO is unlikely to be successful. Intensive Care Med.

[17] Wodchis WP, Bushmeneva K, Nikitovic M, McKillop I (2013). Guidelines on person level cost using administrative databases in Ontario.

[18] Iguchi A, Ridout DA, Galan S, Bodlani C, Squire K, O’Callaghan M, Brown KL (2013). Long-term survival outcomes and causes of late death in neonates, infants, and children treated with extracorporeal life support. Pediatr Crit Care Med.

[19] Almond CS, Singh TP, Gauvreau K, Piercey GE, Fynn-Thompson F, Rycus PT, Bartlett RH, Thiagarajan RR (2011). Extracorporeal membrane oxygenation for bridge to heart transplantation among children in the United States: analysis of data from the Organ Procurement and Transplant Network and Extracorporeal Life Support Organization Registry. Circulation.

[20] Dipchand AI, Mahle WT, Tresler M, Naftel DC, Almond C, Kirklin JK, Pruitt E, Webber SA (2015). Extracorporeal membrane oxygenation as a bridge to pediatric heart transplantation: effect on post-listing and post-transplantation outcomes. Circ Heart Fail.

[21] Lowry AW, Morales DL, Graves DE, Knudson JD, Shamszad P, Mott AR, Cabrera AG, Rossano JW (2013). Characterization of extracorporeal membrane oxygenation for pediatric cardiac arrest in the United States: analysis of the kids’ inpatient database. Pediatr Cardiol.

[22] Roberts TE (1998). Economic evaluation and randomised controlled trial of extracorporeal membrane oxygenation: UK collaborative trial. The Extracorporeal Membrane Oxygenation Economics Working Group. BMJ.

[23] Vats A, Pettignano R, Culler S, Wright J (1998). Cost of extracorporeal life support in pediatric patients with acute respiratory failure. Crit Care Med.

[24] Harvey MJ, Gaies MG, Prosser LA (2015). U.S. and international in-hospital costs of extracorporeal membrane oxygenation: a systematic review. Appl Health Econ Health Policy.

[25] Faraoni D, Nasr VG, DiNardo JA, Thiagarajan RR (2016). Hospital costs for neonates and children supported with extracorporeal membrane oxygenation. J Pediatr.

[26] Johnson JT, Wilkes JF, Menon SC, Tani LY, Weng HY, Marino BS, Pinto NM (2018). Admission to dedicated pediatric cardiac intensive care units is associated with decreased resource use in neonatal cardiac surgery. J Thorac Cardiovasc Surg.

[27] Graves JM, Kannan N, Mink RB, Wainwright MS, Groner JI, Bell MJ, Giza CC, Zatzick DF, Ellenbogen RG, Boyle LN (2016). Guideline adherence and hospital costs in pediatric severe traumatic brain injury. Pediatr Crit Care Med.

[28] Reardon PM, Fernando SM, Van Katwyk S, Thavorn K, Kobewka D, Tanuseputro P, Rosenberg E, Wan C, Vanderspank-Wright B, Kubelik D (2018). Characteristics, outcomes, and cost patterns of high-cost patients in the intensive care unit. Crit Care Res Pract.

[29] Anesi GL, Admon AJ, Halpern SD, Kerlin MP (2019). Understanding irresponsible use of intensive care unit resources in the USA. Lancet Respir Med.

[30] Kyeremanteng K, Downar J (2019). Why is it so hard to stop doing things that are unwanted, non-beneficial, or unsustainable?. Lancet Respir Med.

[31] Schmidt M, Burrell A, Roberts L, Bailey M, Sheldrake J, Rycus PT, Hodgson C, Scheinkestel C, Cooper DJ, Thiagarajan RR (2015). Predicting survival after ECMO for refractory cardiogenic shock: the survival after veno-arterial-ECMO (SAVE)-score. Eur Heart J.

[32] Schmidt M, Bailey M, Sheldrake J, Hodgson C, Aubron C, Rycus PT, Scheinkestel C, Cooper DJ, Brodie D, Pellegrino V (2014). Predicting survival after extracorporeal membrane oxygenation for severe acute respiratory failure. The Respiratory Extracorporeal Membrane Oxygenation Survival Prediction (RESP) score. Am J Respir Crit Care Med.

[33] Hodgson CL, Hayes K, Everard T, Nichol A, Davies AR, Bailey MJ, Tuxen DV, Cooper DJ, Pellegrino V (2012). Long-term quality of life in patients with acute respiratory distress syndrome requiring extracorporeal membrane oxygenation for refractory hypoxaemia. Crit Care.

[34] Garcia Guerra G, Zorzela L, Robertson CM, Alton GY, Joffe AR, Moez EK, Dinu IA, Ross DB, Rebeyka IM, Lequier L (2015). Survival and neurocognitive outcomes in pediatric extracorporeal-cardiopulmonary resuscitation. Resuscitation.

[35] Garcia Guerra G, Robertson CM, Alton GY, Joffe AR, Moez EK, Dinu IA, Ross DB, Rebeyka IM, Lequier L (2014). Health-related quality of life in pediatric cardiac extracorporeal life support survivors. Pediatr Crit Care Med.

[36] Brodie D, Bacchetta M (2011). Extracorporeal membrane oxygenation for ARDS in adults. N Engl J Med.

[37] Abrams D, Combes A, Brodie D (2014). Extracorporeal membrane oxygenation in cardiopulmonary disease in adults. J Am Coll Cardiol.

[38] Barbaro RP, Odetola FO, Kidwell KM, Paden ML, Bartlett RH, Davis MM, Annich GM (2015). Association of hospital-level volume of extracorporeal membrane oxygenation cases and mortality. Analysis of the extracorporeal life support organization registry. Am J Respir Crit Care Med.

[39] IJsselstijn H, Hunfeld M, Schiller RM, Houmes RJ, Hoskote A, Tibboel D, van Heijst AFJ (2018). Improving long-term outcomes after extracorporeal membrane oxygenation: from observational follow-up programs toward risk stratification. Front Pediatr.

[40] Yarnell CJ, Fu L, Manuel D, Tanuseputro P, Stukel T, Pinto R, Scales DC, Laupacis A, Fowler RA (2017). Association between immigrant status and end-of-life care in Ontario, Canada. JAMA.

