# Supplementary material for: Long-term survival and costs following extracorporeal membrane oxygenation in critically ill children—a population-based cohort study
Source: Crit Care. 2020 Apr 6;24:131. doi: 10.1186/s13054-020-02844-3 (PMC7137509; doi:10.1186/s13054-020-02844-3)
Supplement: Supplementary file 1 — Additional file 1 : Supplemental Table 1. Relevant databases and codes utilized for ECMO identification, and International Classification of Diseases, Version 10 (ICD-10) diagnostic codes for categorization. [file 13054_2020_2844_MOESM1_ESM.docx]

**Supplemental Table 1:** Relevant databases and codes utilized for ECMO identification, and International Classification of Diseases, Version 10 (ICD-10) diagnostic codes for categorization.

| **Definition** | **Associated database and codes** |
| --- | --- |
| ECMO Inpatient intervention flag | Database:  Discharge Abstract Database  Codes:  1LZ37GPQM (installation)  1LZ37HHGB (installation)  1LZ37LAQM (installation)  1LZ37GPGB (installation)  1LZ38JAGB (management)  1LZ38JAGC (management)  1LZ38JAQM (management) |
| ECMO physician billing flag | Database:  Ontario Health Insurance Plan  Codes:  Z788 |
| ECMO Indication **based on most responsible diagnosis for admission*    Respiratory failure      Cardiac failure  Other | Database:  Discharge Abstract Database: ICD-10 Codes  P285, Q3212, P220, Q336, P229, P230, R068, J80, B400, B402, B402, B440, B59, J09, J100, J101, J108, J111, J121, J13, J14, J151, J152, J154 ,J159, J168, J180, J181, J188, J189, J209, J210, J22, J398, J438, J439, J440, J441, J448, J449, J4590, J4591, J47, J6799, J680, J690, J701, J708, J80, J82, J841, J848, J849, J850, J851, J869, J90, J9588, J960, J9600, J9601, J9609, J9611, J9619, J969, J9690, J9699, J980, J984, J988, J989  Q234, I270, Q213, Q262, Q2038, Q212, Q245, Q249, P071, Q206, T820, I471, I99, P240, P293, Q201, Q2031, Q210, Q225, Q233, Q254, T811, K721, P2918, Q200, Q204, Q211, Q230, Q231, Q248, I652, I788, I81, I822, I971, K559, K565, K598, P251, P290, Q2058, Q220, Q224, Q232, Q239, Q242, Q893, T817, T822, I460, I469, I472, I4900, I210, I211, I212, I213, I214, I219, I240, I249, I2510, I2511, I2515, I2519, I255, I400, I408, I409,I420, I422, I425, I428, I429, I500, I501, I509,I514, R570  T810, T828, Z540, G931, M4114, M4194, M4195, Q790, C910, G936, I288, I371, I632, I776, P284, P360, P362, P504, P769, R633, T286, Z048, Z488, Z539, A400, A403, A409, A410, A412, A414, A4150, A4158, A4188, A419, A481, A483, A491, A499, R572, |
