# Supplementary material for: Long-term survival and costs following extracorporeal membrane oxygenation in critically ill children—a population-based cohort study
Source: Crit Care. 2020 Apr 6;24:131. doi: 10.1186/s13054-020-02844-3 (PMC7137509; doi:10.1186/s13054-020-02844-3)
Supplement: Supplementary file 2 — Additional file 2 : Supplemental Table 2. Ontario Health Insurance Plan (OHIP), International Classification of Diseases, Version 9 (ICD-9), and Version 10 (ICD-10) diagnostic codes for categorization of comorbidities. [file 13054_2020_2844_MOESM2_ESM.docx]

**Supplemental Table 2:** Ontario Health Insurance Plan (OHIP), International Classification of Diseases, Version 9 (ICD-9), and Version 10 (ICD-10) diagnostic codes for categorization of comorbidities.

| **Condition [reference for validated algorithm]** | **ICD 9 / OHIP** | **ICD 10** | **ODB*** |
| --- | --- | --- | --- |
| Acute Myocardial Infarction (AMI) [1] | 410 | I21, I22 |  |
| Osteo- and other Arthritis:   1. Osteoarthritis 2. Other Arthritis (includes Synovitis, Fibrositis, Connective tissue disorders, Ankylosing spondylitis, Gout Traumatic arthritis, pyogenic arthritis, Joint derangement, Dupuytren’s contracture, Other MSK disorders) | 715  727, 729, 710, 720, 274, 716, 711,718, 728, 739 | M15-M19  M00-M03, M07, M10, M11-M14, M20-M25, M30-M36,  M65-M79 |  |
| Arthritis - Rheumatoid arthritis [2] | 714 | M05-M06 |  |
| Asthma [3] | 493 | J45 |  |
| (all) Cancers | 140-239 | C00-C26, C30-C44, C45-C97 |  |
| Cardiac Arrhythmia | 427 (OHIP) / 427.3 (DAD) | I48.0, I48.1 |  |
| Congestive Heart Failure [4] | 428 | I500, I501, I509 |  |
| Chronic Obstructive Pulmonary Disease [5] | 491, 492, 496 | J41, J43, J44 |  |
| Coronary syndrome (excluding AMI) | 411-414 | I20, I22-I25 |  |
| Dementia [6] | 290, 331 (OHIP) / 046.1, 290.0, 290.1,  290.2, 290.3, 290.4, 294, 331.0, 331.1, 331.5, F331.82 (DAD) | F00, F01, F02, F03, G30 | Cholinesterase  Inhibitors |
| Diabetes [7] | 250 | E08-E13 |  |
| Hypertension [8] | 401, 402, 403, 404, 405 | I10, I11, I12, I13, I15 |  |
| Inflammatory Bowel Disease (IBD) [9] | 555,556 | K50, K51 |  |
| (Other) Mental Illnesses | 291, 292, 295, 297, 298, 299, 301, 302, 303, 304, 305, 306, 307, 313, 314, 315, 319 | F04, F050, F058, F059, F060, F061, F062, F063, F064, F07, F08, F10, F11, F12, F13, F14, F15, F16, F17, F18, F19, F20,  F21, F22, F23, F24, F25, F26, F27, F28, F29, F340, F35, F36,  F37, F430, F439, F453, F454, F458, F46, F47, F49, F50, F51,  F52, F531, F538, F539, F54, F55, F56, F57, F58, F59, F60,  F61, F62, F63, F64, F65, F66, F67, F681, F688, F69, F70,  F71, F72, F73, F74, F75, F76, F77, F78, F79, F80, F81, F82,  F83, F84, F85, F86, F87, F88, F89, F90, F91, F92, F931,  F932, F933, F938, F939, F94, F95, F96, F97, F98 |  |
| Mood, anxiety, depression and other nonpsychotic disorders | 296, 300, 309, 311 | F30, F31, F32, F33, F34 (excl. F34.0), F38, F39, F40, F41,  F42, F43.1, F43.2, F43.8, F44, F45.0, F45.1, F45.2, F48,  F53.0, F68.0, F93.0, F99 |  |
| Osteoporosis | 733 | M81, M82 |  |
| Renal failure | 403, 404, 584, 585, 586, v451 | N17, N18, N19, T82.4, Z49.2, Z99.2 |  |
| Stroke (excluding transient ischemic attack) | 430, 431, 432, 434, 436 | I60I64 |  |

NOTES:

- Abbreviations: DAD=Discharge Abstract Database; ICD = International Classification of Disease; ODB = Ontario Drug Benefit Claims database; OHIP = Ontario Health Insurance Plan Claims Database
- All available health administrative data (OHIP, DAD, ODB) prior to index is used to ascertain disease status, with the exception of AMI (1 year prior to index), Cancer (2 years), Mood Disorder (2 years) and Other Mental Illnesses (2 years) as these conditions are considered episodic
- *ODB prescription drug records are not available for the majority of persons under the age of 65
- AMI, Asthma, COPD, CHF, Dementia, Diabetes, Hypertension, IBD, and Rheumatoid Arthritis are based on validated case algorithms/ ICES cohorts (see 1-9 below, respectively). All other conditions required at least one diagnosis code recorded in acute care (DAD) or two diagnosis codes recorded in physician billings (OHIP) within a two-year period.

References:

1. Austin PC, Daly PA, Tu JV. A multicenter study of the coding accuracy of hospital discharge administrative data for patients admitted to cardiac care units in Ontario. American Heart

Journal 2002;144:290–6.

2. Widdifield J, Bernatsky S, Paterson JM, Tu K, Ng R, Thorne JC, Pope JE, Bombardier C. Accuracy of Canadian health administrative databases in identifying patients with rheumatoid

arthritis: a validation study using the medical records of rheumatologists. Arthritis Care Res 2013; 65(10): 1582-1591.

3. Gershon AS, Wang C, Guan J, Vasilevska-Ristovska J, Cicutto L, To T. Identifying patients with physician-diagnosed asthma in health administrative databases. Can Respir J 2009;16:183–8.

4. Schultz SE, Rothwell DM, Chen Z, Tu K. Identifying cases of congestive heart failure from administrative data: a validation study using primary care patient records. Chronic Diseases and

Injuries in Canada 2013;33:160–6.

5. Gershon AS, Wang C, Guan J, Vasilevska-Ristovska J, Cicutto L, To T. Identifying Individuals with Physician Diagnosed COPD in Health Administrative Databases. Copd 2009;6:388–94.

6. Jaakkimainen RL, Bronskill SE, Tierney MC, Herrmann N, Green D, Young J, et al. Identification of Physician-Diagnosed Alzheimer’s Disease and Related Dementias in Population-Based Administrative Data: A Validation Study Using Family Physicians’ Electronic Medical Records. J Alzheimers Dis.; 2016 Aug 10;54(1):337–49

7. Hux JE, Ivis F, Flintoft V, Bica A. Diabetes in Ontario: Determination of prevalence and incidence using a validated administrative data algorithm. Diabetes Care 2002;25:512–6.

8. Tu K, Campbell NR, Chen ZL, Cauch-Dudek KJ, McAlister FA. Accuracy of administrative databases in identifying patients with hypertension. Open Med 2007;1:e18–26.

9. Benchimol EI, Guttmann A, Mack DR, Nguyen GC, Marshall JK, Gregor JC, Wong J, Forster AJ, Manuel D. Validation of international algorithms to identify adults with inflammatory bowel disease in health administrative data from Ontario, Canada, J Clin Epidemiol. 2014; 67(8):887-96
