# Supplementary material for: Long-term survival and costs following extracorporeal membrane oxygenation in critically ill children—a population-based cohort study
Source: Crit Care. 2020 Apr 6;24:131. doi: 10.1186/s13054-020-02844-3 (PMC7137509; doi:10.1186/s13054-020-02844-3)
Supplement: Supplementary file 3 — Additional file 3 : Supplemental Table 3. Hierarchy approach for discharge disposition of those who survived to discharge. [file 13054_2020_2844_MOESM3_ESM.docx]

**Supplemental Table 3:** Hierarchy approach for discharge disposition of those who survived to discharge

Hierarchy as follows:

1. **Discharged to home with homecare (**defined as: at least one homecare service within 21 days of discharge, as identified via the Home Care Database)
2. **Discharged to rehabilitation or complex continuing care** (defined as: at least one admission to complex continuing care or a rehabilitation bed, as identified via the Continuing Care Reporting System or the National Rehabilitation Reporting System, respectively)
3. **Discharged to a long-term care facility** (defined as: at least one admission to long-term care within 2 days of discharge, as identified via the Continuing Care Reporting System – Long-term Care)
4. **Died in hospital** (defined as: death date occurring on or prior to discharge date, as identified via the Discharge Abstract Database)
5. **Discharged to home without homecare** (if patient did not meet other four criteria above, then included in this group)

*In cases where a patient is eligible for more than one disposition category, the institution with the first date is selected as priority. In cases where the dates overlap, the following hierarchy is used: death > complex continuing care/rehabilitation > long-term care facility > homecare
