# Supplementary material for: Long-term survival and costs following extracorporeal membrane oxygenation in critically ill children—a population-based cohort study
Source: Crit Care. 2020 Apr 6;24:131. doi: 10.1186/s13054-020-02844-3 (PMC7137509; doi:10.1186/s13054-020-02844-3)
Supplement: Supplementary file 4 — Additional file 4 : Supplemental Table 4. Most common “Most Responsible Diagnoses” among included patients. [file 13054_2020_2844_MOESM4_ESM.docx]

**Supplemental Table 4:** Most common “Most Responsible Diagnoses” among included patients, as based upon International Classification of Diseases, Version 10 (ICD-10) codes.

| **Most Responsible Diagnosis** | **Number of Patients (%)** |
| --- | --- |
| **Neonatal, *n* = 103** | |
| Hypoplastic Left Heart Syndrome | 27 (26.2) |
| Complete Transposition of Great Vessels | 14 (13.6) |
| Congenital Diaphragmatic Hernia | 13 (12.6) |
| Tetralogy of Fallot | 13 (12.6) |
| Truncus Arteriosus | 11 (10.7) |
| Double Inlet Ventricle | 10 (9.7) |
| **Cardiac Failure, *n* = 169** | |
| Hypoplastic Left Heart Syndrome | 29 (17.2) |
| Atrioventricular Septal Defect | 24 (14.2) |
| Tetralogy of Fallot | 22 (13.0) |
| Dilated Cardiomyopathy | 12 (7.1) |
| Double Outlet Right Ventricle | 12 (7.1) |
| Total Anomalous Pulmonary Venous Connection | 12 (7.1) |
| **Respiratory Failure, *n* = 70** | |
| Sepsis/Septic Shock | 15 (21.4) |
| Respiratory Distress Syndrome | 10 (14.3) |
| Bacterial Pneumonia | 6 (8.6) |
