# Supplementary material for: Long-term survival and costs following extracorporeal membrane oxygenation in critically ill children—a population-based cohort study
Source: Crit Care. 2020 Apr 6;24:131. doi: 10.1186/s13054-020-02844-3 (PMC7137509; doi:10.1186/s13054-020-02844-3)
Supplement: Supplementary file 5 — Additional file 5 : Supplemental Table 5. Comparison of neonatal patients receiving Extra Corporeal Membrane Oxygenation who survive to hospital discharge against those who died in-hospital (n = 342). *≤ 5 patients. aRange provided due to small cell sizes. Abbreviations: SD = standard deviation; ECMO = Extracorporeal Membrane Oxygenation; IQR = interquartile range. [file 13054_2020_2844_MOESM5_ESM.docx]

**Supplemental Table 5:** Comparison of neonatal patients receiving Extra Corporeal Membrane Oxygenation who survive to hospital discharge against those who died in-hospital (*n =* 342). *≤ 5 patients. ^a^Range provided due to small cell sizes. ^b^Adapted from Feudtner *et al*., *BMC Pediatr*, 2014. Abbreviations: SD = standard deviation; ECMO = Extracorporeal Membrane Oxygenation; IQR = interquartile range

|  | **Overall Cohort** | | |
| --- | --- | --- | --- |
| **Variable** | **Surviving to Discharge**  **(*n* = 55)** | **Died In-hospital**  **(*n* = 48)** | ***P* Value** |
| **Sex, *n* (%)** | | |  |
| Male | 34 (61.8) | 16 (33.3) | 0.61 |
| Female | 21 (38.2) | 32 (66.7) |  |
| **Age, days, mean (SD)** | 8.4 (7.) | 10.9 (8.2) | 0.09 |
| **Income, *n* (%)** | | |  |
| Lowest | 17 (30.9) | 16 (33.3) | 0.11 |
| Low | 9 (16.4) | 12 (25.0) |  |
| Middle | 15 (27.3) | 7 (14.6) |  |
| High | 14 (25.5) | 13 (27.1) |  |
| **Rurality, *n* (%)** | | | 0.66 |
| Urban | 49 (89.1) | 43-48^a^ |  |
| Rural | 6 (10.9) | * |  |
| **Chronic Complex Conditions, *n* (%)^b^** | | | |
| Any Chronic Complex Condition | 41 (74.5) | 40 (83.3) | 0.28 |
| Prematurity | 7 (12.7) | 11 (22.9) | 0.17 |
| Cardiovascular | 32 (58.2) | 29 (60.4) | 0.81 |
| Other Congenital or Genetic Abnormality | * | 10-15^a^ | 0.03 |
| **Time to ECMO from Admission, days, median (IQR)** | 4 (1-8) | 7 (3-13) | 0.03 |
