# Supplementary material for: Long-term survival and costs following extracorporeal membrane oxygenation in critically ill children—a population-based cohort study
Source: Crit Care. 2020 Apr 6;24:131. doi: 10.1186/s13054-020-02844-3 (PMC7137509; doi:10.1186/s13054-020-02844-3)
Supplement: Supplementary file 6 — Additional file 6: Supplemental Table 6. Comparison of patients with cardiac failure receiving Extra Corporeal Membrane Oxygenation who survive to hospital discharge against those who died in-hospital (n = 169). *≤ 5 patients. aRange provided due to small cell sizes. Abbreviations: SD = standard deviation; ECMO = Extracorporeal Membrane Oxygenation; IQR = interquartile range. [file 13054_2020_2844_MOESM6_ESM.docx]

**Supplemental Table 6:** Comparison of patients with cardiac failure receiving Extra Corporeal Membrane Oxygenation who survive to hospital discharge against those who died in-hospital (*n =* 169). *≤ 5 patients. ^a^Range provided due to small cell sizes. ^b^Adapted from Feudtner *et al*., *BMC Pediatr*, 2014. Abbreviations: SD = standard deviation; ECMO = Extracorporeal Membrane Oxygenation; IQR = interquartile range

|  | **Overall Cohort** | | |
| --- | --- | --- | --- |
| **Variable** | **Surviving to Discharge**  **(*n* = 102)** | **Died In-hospital**  **(*n* = 67)** | ***P* Value** |
| **Sex, *n* (%)** | | | 0.30 |
| Male | 51 (50.0) | 28 (41.8) |  |
| Female | 51 (50.0) | 39 (58.2) |  |
| **Age, years, mean (SD)** | 2.4 (4.3) | 3.0 (5.2) | 0.39 |
| **Income, *n* (%)** | | | 0.81 |
| Lowest | 25 (24.5) | 12 (17.9) |  |
| Low | 20 (19.6) | 16 (23.9) |  |
| Middle | 23 (22.5) | 12 (17.9) |  |
| High | 18 (17.6) | 15 (22.4 |  |
| Highest | 14 (13.7) | 11 (16.4) |  |
| Unknown | * | * |  |
| **Rurality, *n* (%)** | | | 0.51 |
| Urban | 89 (87.3) | 59 (88.1) |  |
| Rural | 11 (10.8) | 8 (11.9) |  |
| **Chronic Complex Conditions, *n* (%)^b^** | | | |
| Any Chronic Complex Condition | 81 (79.4) | 59 (88.1) | 0.15 |
| Prematurity | 12 (11.8) | 11 (16.4) | 0.39 |
| Cardiovascular | 77 (75.5) | 55 (82.1) | 0.31 |
| Other Congenital or Genetic Abnormality | 21 (20.6) | 10 (14.9) | 0.35 |
| **Time to ECMO from Admission, days, median (IQR)** | 3 (0-7) | 11 (3-35) | <0.001 |
| **Ventricular Assist Device, *n* (%)** | 11 (10.8) | 18 (26.9) | <0.01 |
| **Heart Transplant, *n* (%)** | 7 (6.9) | 6 (9.0) | 0.62 |
