# Supplementary material for: Long-term survival and costs following extracorporeal membrane oxygenation in critically ill children—a population-based cohort study
Source: Crit Care. 2020 Apr 6;24:131. doi: 10.1186/s13054-020-02844-3 (PMC7137509; doi:10.1186/s13054-020-02844-3)
Supplement: Supplementary file 7 — Additional file 7 : Supplemental Table 7. Comparison of patients with respiratory failure receiving Extra Corporeal Membrane Oxygenation who survive to hospital discharge against those who died in-hospital (n = 70). *≤ 5 patients. aRange provided due to small cell sizes. Abbreviations: SD = standard deviation; ECMO = Extracorporeal Membrane Oxygenation; IQR = interquartile range. [file 13054_2020_2844_MOESM7_ESM.docx]

**Supplemental Table 7:** Comparison of patients with respiratory failure receiving Extra Corporeal Membrane Oxygenation who survive to hospital discharge against those who died in-hospital (*n =* 70). *≤ 5 patients. ^a^Range provided due to small cell sizes. ^b^Adapted from Feudtner *et al*., *BMC Pediatr*, 2014. Abbreviations: SD = standard deviation; ECMO = Extracorporeal Membrane Oxygenation; IQR = interquartile range

|  | **Respiratory Failure** | | |
| --- | --- | --- | --- |
| **Variable** | **Surviving to Discharge**  **(*n* = 36)** | **Died In-hospital**  **(*n* = 34)** | ***P* Value** |
| **Sex, *n* (%)** | | | 0.77 |
| Male | 21 (58.3) | 21 (61.8) |  |
| Female | 15 (41.7) | 13 (38.2) |  |
| **Age, years, mean (SD)** | 7.7 (6.4) | 6.3 (6.0) | 0.42 |
| **Income, *n* (%)** | | | 0.19 |
| Lowest | 6 (16.7) | 11 (32.4) |  |
| Low | * | * |  |
| Middle | 6 (16.7) | 9 (26.5) |  |
| High | 13 (36.1) | 6 (17.6) |  |
| Highest | * | * |  |
| **Rurality, *n* (%)** | | | 0.44 |
| Urban | 31-36^a^ | 29-34^a^ |  |
| Rural | * | * |  |
| **Chronic Complex Conditions, *n* (%)^b^** | | | |
| Any Chronic Complex Condition | 24 (66.7) | 29 (82.9) | 0.11 |
| Prematurity | 6-11^a^ | * | 0.11 |
| Cardiovascular | 11 (30.6) | 13 (37.1) | 0.55 |
| Other Congenital or Genetic Abnormality | * | 5-10^a^ | 0.47 |
| **Time to ECMO from Admission, days, median (IQR)** | 3 (0-15) | 9 (1-25) | 0.12 |
| **Lung Transplant, *n* (%)** | 7 (19.4) | * | 0.20 |
