# Supplementary material for: Long-term survival and costs following extracorporeal membrane oxygenation in critically ill children—a population-based cohort study
Source: Crit Care. 2020 Apr 6;24:131. doi: 10.1186/s13054-020-02844-3 (PMC7137509; doi:10.1186/s13054-020-02844-3)
Supplement: Supplementary file 8 — Additional file 8 : Supplemental Table 8. Mean (standard deviation) 1-year costs following admission of pediatric patients requiring Extra Corporeal Membrane Oxygenation in Ontario, Canada (2009-2016). All values in Canadian dollars. Abbreviations: SD = standard deviation; OHIP = Ontario Health Insurance Plan. [file 13054_2020_2844_MOESM8_ESM.docx]

**Supplemental Table 8:** Mean (standard deviation) 1-year costs following admission of pediatric patients requiring Extra Corporeal Membrane Oxygenation in Ontario, Canada (2009-2016). All values in Canadian dollars. Abbreviations: SD = standard deviation; OHIP = Ontario Health Insurance Plan

| **Cost Sector** | **Overall**  **(*n* = 342)** | **Neonatal**  **(*n* = 103)** | **Cardiac Failure**  **(*n* = 169)** | **Respiratory Failure**  **(*n* = 70)** | ***P* Value** |
| --- | --- | --- | --- | --- | --- |
| **Acute Care Sectors** |  | | | | |
| Inpatient | 199,926  (269,476) | 169,243  (157,408) | 215,026  (219,571) | 208,620  (450,362) | 0.38 |
| Emergency Department | 541  (790) | 493  (872) | 523  (723) | 658  (818) | 0.37 |
| **Continuing Care Sectors** | | | | | |
| Complex Continuing Care | 88  (1,630) | 0  (0) | 178  (2,319) | 0  (0) | 0.60 |
| Long-term Care | 0  (0) | 0  (0) | 0  (0) | 0  (0) | 0.60 |
| Rehabilitation | 0  (0) | 0  (0) | 0  (0) | 0  (0) | 1.0 |
| Home Care | 4,554  (18,842) | 1,445  (3,126) | 3,984  (16,696) | 10,504  (31,787)) | <0.01 |
| **Outpatient Care Sectors** | | | | | |
| Outpatient Clinics | 1,926  (2,927) | 1,753  (2,375) | 1,794  (2,851) | 2,500  (3,715) | 0.18 |
| Laboratory (OHIP) | 11  (48) | 4  (16) | 25  (77) | 10  (48) | 0.02 |
| Drugs (Ontario Drug  Benefit Program) | 1,153  (4,019) | 269  (542) | 1,295  (4,409) | 2,111  (5,468) | 0.01 |
| **Physician Billings** | 23,894  (18,597) | 21,753  (15,418) | 25,514  (18,824) | 23,133  (21,937) | 0.25 |
| **Total Costs** | **232,705**  **(285,830)** | **195,113**  **(172,022)** | **248,834**  **(138,780)** | **249,083**  **(467,091)** | 0.28 |
